# Prevalence of sarcopenia in Chinese community-dwelling elderly: a systematic review

**DOI:** 10.1186/s12889-022-13909-z

**Published:** 2022-09-08

**Authors:** Xiaoyu Ren, Xianliang Zhang, Qiang He, Litao Du, Ke Chen, Si Chen, Yang Pan

**Affiliations:** 1 School of Physical Education, Shandong University, 17922 Jingshi Road, Lixia District, Shandong Province 250061 Jinan City, China; 2 School of Nursing and Rehabilitation, Cheeloo College of Medicine, Shandong University, 44 West Wenhua Road, Lixia District, Shandong Province 250102 Jinan City, China

**Keywords:** Sarcopenia, Prevalence, Older Adults, China, Meta-analysis

## Abstract

**Background:**

Sarcopenia is associated with age-related loss of muscle mass and function and is becoming prevalent in the older Chinese population. This systematic review aims to obtain a reliable estimation of the prevalence of sarcopenia among community-dwelling Chinese populations aged 65 years and older and to characterize its epidemiology.

**Methods:**

A literature search was performed in the Cochrane Library, PubMed, Web of Science, China National Knowledge Infrastructure (CNKI), Wanfang Data, and CQVIP databases up to September 31, 2021. All studies that reported the prevalence of sarcopenia in Chinese community-dwelling older adults were included, and Hoy et al.’s tool was used to assess the risk of bias. The overall prevalence of sarcopenia will be calculated as the primary outcome, and subgroup analyses will be performed by study year, age, sex, muscle mass assessment method, diagnostic criteria and area.

**Results:**

A total of 26 studies were included in this study, which involved 25,921 subjects, and 3597 had sarcopenia. Although significant heterogeneity between studies was reported, no statistically significant publication bias was detected. The overall prevalence of sarcopenia in community-dwelling older adults aged over 65 years in the Chinese population was 17.4% (95% CI: 14.6%-20.2%). Subgroup analysis based on study year, age and sex, muscle mass assessment method, diagnostic criteria, region and area showed that the prevalence of sarcopenia was different in each subgroup.

**Implications:**

The prevalence of sarcopenia in Chinese community-dwelling older adults was higher than that in previous studies. As a multidimensional survey of the prevalence of sarcopenia in older adults, this meta-analysis provides data support for the targeted management of sarcopenia among Chinese older adults.

**Supplementary Information:**

The online version contains supplementary material available at 10.1186/s12889-022-13909-z.

## Introduction

Sarcopenia is characterized by a progressive loss of skeletal muscle mass, strength and performance [1]. Although it has been included in the 10th edition of the International Classification of Diseases Clinical Modification (ICD-10-CM) with a disease code M62.84 by the World Health Organization (WHO), it is still a common but low level of awareness disease among older adults. In this global aging environment, sarcopenia is associated with many age-related chronic diseases, which may increase the incidence of falls in older adults [2], fractures [3], functional limitation and physical disability [4], and all-cause mortality [5], contributing to poor quality of life. As a previous systematic review reported, sarcopenia affects 9.9%-40.4% of community-dwelling older adults worldwide [6], although some estimates are as high as 60% [7]. These great variations in the prevalence of sarcopenia might be primarily explained by different diagnostic criteria for sarcopenia.

Several guidelines have been published for the early identification, diagnosis and management of sarcopenia. The European Working Group on Sarcopenia (EWGSOP) introduced the first consensus diagnostic criteria for sarcopenia in 2010 [8]. The EWGSOP recommended using the presence of both low muscle mass and low muscle function (strength or performance) for sarcopenia diagnosis and provided the cutoff points of muscle mass, muscle strength, and physical performance with slowing walking speed (≤ 0.8 m/s) or low grip strength (< 20 kg for women and < 30 kg for men, respectively), indicating the onset of sarcopenia. In 2011, the International Working Group on Sarcopenia (IWGS) published the second consensus definition for sarcopenia [9]. The IWGS suggested the diagnosis of sarcopenia as a gait speed less than 1 m/s and objectively measured low muscle mass (appendicular skeletal muscle mass relative to square of height (ASM/height^2^) ≤ 7.23 kg/m^2^ in men and ≤ 5.67 kg/m^2^ in women respectively). The American Foundation for the National Institutes of Health (FNIH) also published their official consensus in 2014. Given the differences in ethnicity, genetic background, diet pattern, and body size, these criteria might not be appropriate for Asians [10]. Thus, the Asian Working Group for Sarcopenia (AWGS) established a consensus for sarcopenia diagnosis appropriate to Asians in 2014 [11]. The AWGS agreed with previous reports that sarcopenia should be described as low muscle mass plus low muscle strength and/or low physical performance. The AWGS recommended cutoff values for muscle mass (≤ 7.0 kg/m^2^ for men and 5.4 kg/m^2^ for women by dual X-ray absorptiometry, and ≤ 7.0 kg/m^2^ for men and 5.7 kg/m^2^ by using bioimpedance analysis for women) appropriate for Asians. In 2018, the EWGSOP updated the original definition for sarcopenia to increase the consistency of clinical diagnoses [12]. The EWGSOP2 focused on low muscle strength as a key characteristic of sarcopenia. Specifically, sarcopenia is probable when low muscle strength is detected, and sarcopenia is confirmed by the presence of low muscle quantity or quality. When low muscle strength, low muscle quantity/quality and low physical performance were all detected, sarcopenia was considered severe. To increase the harmonization to sarcopenia studies, the EWGSOP2 recommended cutoff points for low muscle strength (grip strength < 27 kg for men and < 16 kg for women, chair stand > 15 s for five rises), low muscle quantity (ASM < 20 kg for men and < 15 kg for women, ASM/height^2^ < 7.0 kg/m^2^ for men and < 5.5 kg/m^2^ for women), and low physical performance (gait speed ≤ 0.8 m/s, Short Physical Performance Battery (SPPB) ≤ 8 points, Time up and Go test (TUG) ≥ 20 s, 400 m walk test noncompletion or ≥ 6 min for completion). Therefore, the use of different cutoff values, different techniques used for muscle measurement and study methodologies makes it challenging to accurately estimate the burden of this disease.

China is facing a severe aging situation with a rapid growth of the aging population. According to the latest data of the Seventh National Population Census issued in May 2021, the number of people aged 65 and over reached 190,635,280, accounting for 13.50% of the whole national population [13], and this percentage almost reached a deeply aging society (14%). Compared with the results of the Sixth National Population Census conducted in 2010, the proportion increased by 4.63%. To realize the goal of healthy aging, it is essential to evaluate the overall prevalence of sarcopenia in community-dwelling older Chinese adults. While more recent studies have focused on the prevalence of sarcopenia, the results have been inconsistent among studies [10, 14, 15]. Currently, several systematic reviews have performed meta-analyses to estimate the prevalence of sarcopenia in Chinese community-dwelling older adults. Some reported an overall prevalence (12%) [16], with the key point being mainland China (17%) being higher than that in nonmainland areas (6%). Some separately reported a prevalence based on the AWGS (14%), IWGS (18%) and EWGSOP (10%) [17]. However, as a result of the limited numbers of studies included, most included studies were performed in eastern coastal China areas, while western and urban areas were less investigated, which might underestimate the prevalence of sarcopenia. Several large-scale sample studies have been published in the past two years and will be available for this systematic review and meta-analysis. Therefore, a meta-analysis was conducted to evaluate the prevalence of sarcopenia in Chinese community-dwelling older adults to solve the heterogeneity in sarcopenia prevalence among studies.

## Methods

### Protocol and registration

This review was conducted in accordance with the Preferred Reporting Items for Systematic Reviews and Meta Analyses (PRISMA) statement [18]. This review is a prevalence study, so ethics approval was not required for this research. The protocol for this systematic review and meta-analysis was registered at PROSPERO with number CRD42021228612.

### Data sources and searches

The electronic search strategy was developed to search the following electronic bibliographic databases up to March 2021: Cochrane Library, PubMed, Web of Science and China National Knowledge Infrastructure (CNKI), Wanfang Data, and CQVIP. All studies that reported the prevalence of sarcopenia in Chinese community-dwelling older adults were searched without any restrictions of country or article type. The research strategy used the descriptors in English: “prevalence”, “epidemiology”, “incidence”, “sarcopenia”, “aged”, “older people”, “elderly”, “Chinese”, “China” and their combinations. The complete search strategy was as follows: PubMed: (((sarcopenia[MeSH Terms]) OR (sarcopenias[Title/Abstract])) AND (((aged[MeSH Terms]) OR (older people[Title/Abstract])) OR (elderly[Title/Abstract])) AND (((prevalence[MeSH Terms]) OR (epidemiology[Title/Abstract])) OR (incidence[Title/Abstract])); Web of Science: TS = (sarcopenia) AND TS = (prevalence OR epidemiology OR incidence) AND TS = (aged OR older people OR elderly) AND AD = China AND(PY = 2010–2021); CNKI: (SU = sarcopenia) AND (SU = prevalence OR incidence OR epidemiology) AND (SU = elderly OR older people) AND (YE = 2010.1.1–2021.3.31); Wanfang: (SU = sarcopenia) AND (SU = prevalence OR incidence OR epidemiology) AND (SU = elderly OR older adults) and Date: 2010–2021; CQVIP: (M = sarcopenia) AND (M = prevalence OR incidence OR epidemiology) AND (M = elderly OR older adults). All those studies will assess bias risk.

### Study selection

Two independent reviewers (XYR, XLZ) screened the titles and/or abstracts to identify potentially eligible studies, and all the full texts of these potentially eligible articles were assessed for eligibility. If multiple papers came from the same project, we chose the one with the largest sample size, which might be more consistent with the whole project and data extraction rule of this review. Disagreements between them were resolved through discussion with a third reviewer (QH).

### Inclusion and exclusion criteria

Studies were included in which the participants were Chinese community-dwelling older adults aged 65 years or older. Studies were excluded if they were reviews, meeting abstracts, study protocols without data, letters to editors, studies for which the full text could not be obtained, and studies published in languages other than Chinese or English. Other exclusion criteria included participants recruited from hospitals or long-term care facilities and participants with specific diseases or conditions.

### Data extraction and quality assessment

A prepiloted form was used to extract data including the study characteristics (e.g., first author, publication year, study design), characteristics of the target population (e.g., age, area, gender and sample size), diagnostic criteria for sarcopenia, assessment method used for each parameter (muscle mass, muscle strength and muscle performance), cutoff values of each parameter, and sarcopenia prevalence. For each study, prevalence was calculated as the number of people with sarcopenia divided by the whole sample size. We extracted the baseline data from the cohort studies. Data extraction was performed by two independent reviewers (XYR, XLZ), and disagreements were resolved through discussion with a third reviewer (QH). Missing data were requested from study authors.

Two reviewers independently assessed the risk of bias for each eligible study using Hoy et al.’s [19] risk of bias tool specifically developed to assess bias risk in prevalence studies. The tool consists of 10 items addressing four domains of bias that assess the internal and external validity of the study. Each criterion was rated as high or low risk of bias, and an overall judgment of bias risk was then rated as low, moderate and high. Studies were classified as having a low risk of bias when eight or more of the ten questions were answered as “low risk”, a moderate risk of bias when eight or more of the ten questions were answered as “low risk” and a high risk of bias when five or fewer questions were answered as “low risk”. We intended to present the risk of bias and quality scores in a table.

### Data synthesis and analysis

Data were first analyzed through descriptive statistics. The prevalence of sarcopenia was described in percentage terms. Meta-analysis will be conducted if a sufficient quantity of identified studies is comparable. All studies were stratified by sex and age if possible, and the subgroup analysis also included the assessment method, diagnostic criteria and area. Gender was divided into male and female. For all analyses, the study subjects were Chinese community-dwelling older adults. For the cutoff values of the parameter, the method of muscle mass determination was categorized as dual energy X-ray absorptiometry (DEXA), bioelectric impedance analysis (BIA) or anthropometric measures. Muscle strength was measured by handgrip strength. Physical performance was measured by gait speed.

Heterogeneity between the studies was assessed through the I^2^ test, and I^2^ values greater than 50% were considered moderate to high heterogeneity. A random-effects or fixed-effect model was used depending on the degree of heterogeneity. A funnel plot was used to assess publication bias, and funnel plot asymmetry was assessed by Begg’s test and Egger’s test. Subgroup analyses were conducted after removing studies that did not report the necessary data. Sensitivity analysis was used to investigate the impact of the methodological quality of the eligible studies. The overall quality of evidence was summarized using the Grading of Recommendations Assessment, Development and Evaluation (GRADE) system.

## Results

### Study selection and characteristics

**Fig. 1:**
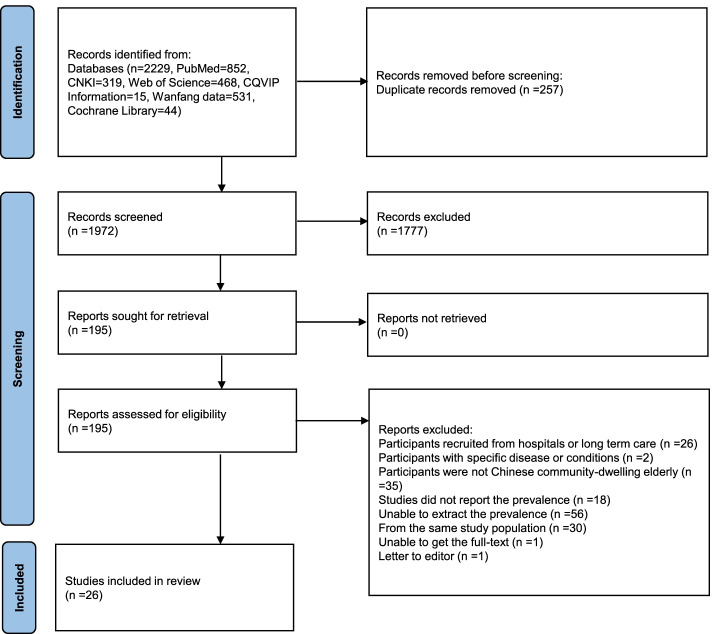
PRISMA flowchart presenting the summary of searches carried out in the literature

The initial searches identified 2229 records. After removing duplicates, 1972 articles were screened for potential eligibility. After title and abstract screening, 1777 records were excluded for different reasons; 195 full texts were assessed using the inclusion criteria, and 26 eligible studies were included in this meta-analysis. The flow diagram for study selection is presented in a PRISMA flow diagram (Fig. 1) as follows:

The characteristics of the 26 included studies are shown in Additional file 1. The studies were published between 2010 and 2021. Of the 26 studies, 8 were conducted in northern China, and 18 were conducted in southern China; 17 and 18 studies focused on males and females, respectively. The sample size varied from 22 to 4576, six studies had a large sample size over 1000 participants [20–25], three studies had a small sample size under 100 participants [26–28], and the total population included in this meta-analysis was 25,921 participants (8742 males and 9685 females). The total number of included participants was not equal to the sum of the numbers of males and females because some studies only provided the total numbers and did not separate participants into males and females. Two studies only included females [28, 29], one study only included males [30], and the others were all mixed gender [20–27, 31–45]. The point prevalence of sarcopenia ranged from 4.48 to 50.84%. The total participants included 2588 aged from 65 to 69 years, 9209 aged from 70 to 79 years, and 3166 aged over 80 years. The total number of included participants was not equal to the sum of participants aged over 65 years, as some studies only provided the total numbers and did not report the number of participants by their age. Sarcopenia was diagnosed mainly according to the AWGS and EWGSOP, and only two used Ishii’s score [46] and the SARC-F [47], respectively. Only one study did not report the assessment method of muscle mass [42].

### Quality assessment

The overall quality of evidence assessed by the GRADE was low, and the risk of bias is shown in Table 1. According to Hoy et al.’s risk of bias tool, 84.6% (*n* = 22) of studies were at moderate risk, 7.7% (*n* = 2) of studies were at low risk and 7.7% (*n* = 2) of studies were at high risk. The overall risk of bias was defined as “moderate risk”.

**Table 1:** Quality assessment of included studies using Hoy’s tool

Study	Q1	Q2	Q3	Q4	Q5	Q6	Q7	Q8	Q9	Q10	Overall risk of bias
Hua et al. [27]	H	H	H	L	L	L	H	L	L	L	M
Wu et al. [34]	H	H	H	L	L	L	L	L	L	L	M
Li et al. [28]	H	H	H	L	L	L	L	L	L	L	M
Wang et al. [41]	H	H	H	L	L	L	L	L	L	H	M
Gao et al. [37]	H	H	H	L	L	L	L	L	L	L	M
Meng et al. [40]	H	H	H	L	L	L	L	L	L	L	M
Chen et al. [33]	H	H	H	L	L	H	L	L	L	L	M
Meng et al. [30]	H	H	H	L	L	L	L	L	L	L	M
Yu et al. [25]	H	H	H	L	L	L	L	L	L	L	M
Xu et al. [44]	H	H	H	L	L	L	L	L	L	L	M
Liu et al. [24]	H	H	H	L	L	L	L	L	L	L	M
Meng et al. [22]	H	H	L	L	L	L	L	L	L	L	L
Deng et al. [20]	H	H	H	L	L	L	L	L	L	L	M
Che et al. [32]	H	H	H	L	L	L	L	L	L	L	M
Jiao et al. [29]	H	H	H	L	L	L	L	L	L	L	M
Du et al. [31]	H	H	H	L	L	L	L	L	L	L	M
Chen et al. [26]	H	H	H	L	L	L	L	L	L	L	M
Kuo et al. [36]	H	H	H	L	L	L	L	L	L	L	M
Xia et al. [43]	H	H	H	L	L	L	L	L	L	L	M
Liu et al. [38]	H	H	H	L	L	L	L	L	L	L	M
Mao et al. [39]	H	H	H	L	L	L	L	L	L	L	H
Huang et al. [35]	H	H	H	L	L	L	L	L	L	L	M
Zhu et al. [45]	H	H	H	L	L	L	H	L	L	L	H
Sun et al. [23]	H	H	H	L	L	L	L	L	L	L	M
Wu et al. [42]	L	H	H	L	L	L	L	L	L	L	L
Lu et al. [21]	H	H	H	L	L	L	L	L	L	L	M

*H* High risk of bias, *M* Moderate risk of bias, *L* Low risk of bias, *Q* Question

### Pooled prevalence of sarcopenia

#### Overall prevalence of sarcopenia

**Fig. 2:**
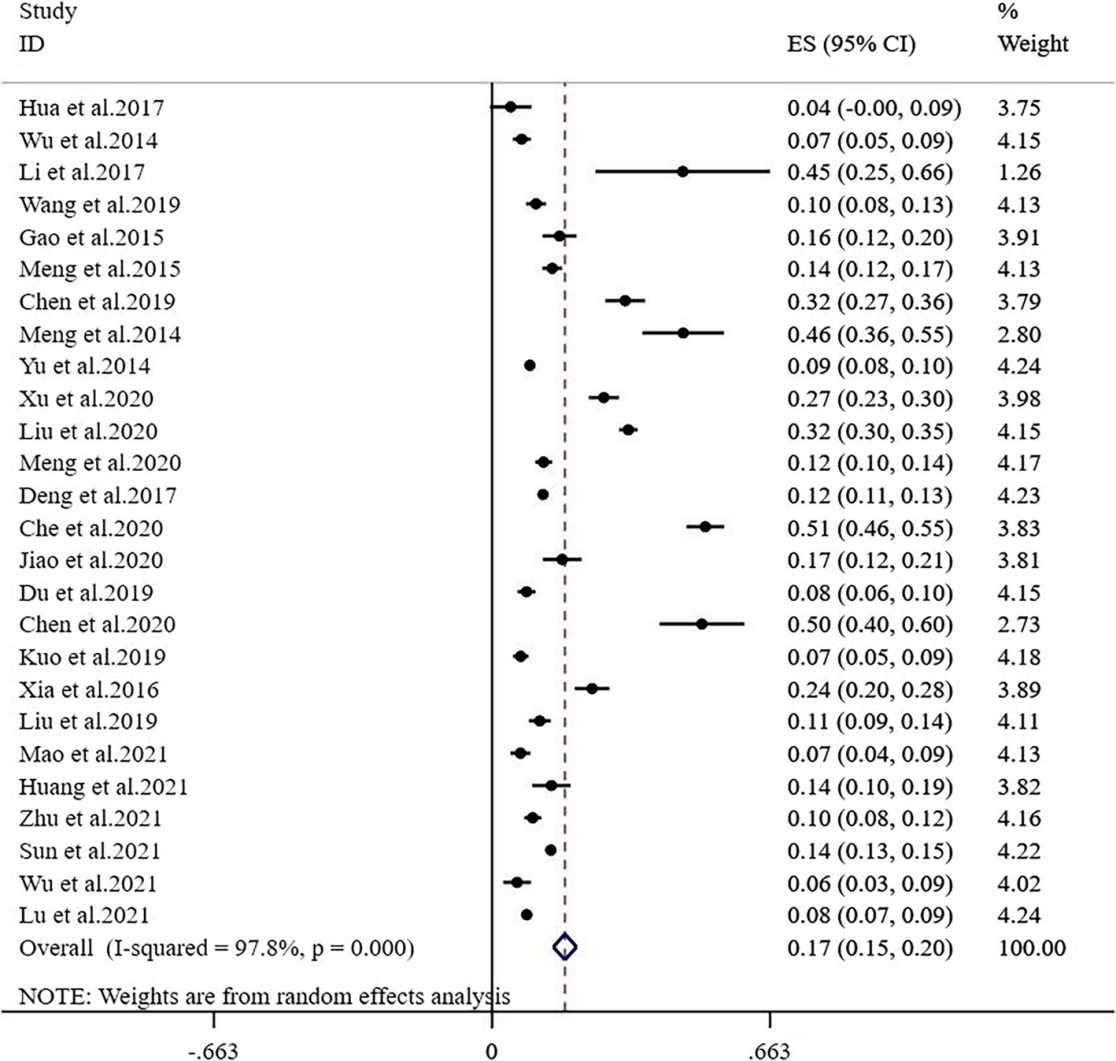
Forest plot of the prevalence of sarcopenia in Chinese community-dwelling older adults

The meta-analysis of the total prevalence estimates of studies showed that the prevalence of sarcopenia in community-dwelling adults aged 65 years and older was 17.4% (95% CI: 14.6%-20.2%), with a high level of heterogeneity (I^2^ = 97.8%, *P* < 0.01) (Fig. 2).

#### Study year

The meta-analysis results showed a general upward trend; the prevalence among studies with data collected from 2014 to 2017 and 2018–2021 was 15.3% (95% CI: 11.9%-18.8%, I^2^ = 94.8%, *P* < 0.01) and 18.0% (95% CI: 13.9%-22.1%, I^2^ = 98.4%, *P* < 0.01), respectively (Additional file 2).

#### Age and gender

**Fig. 3:**
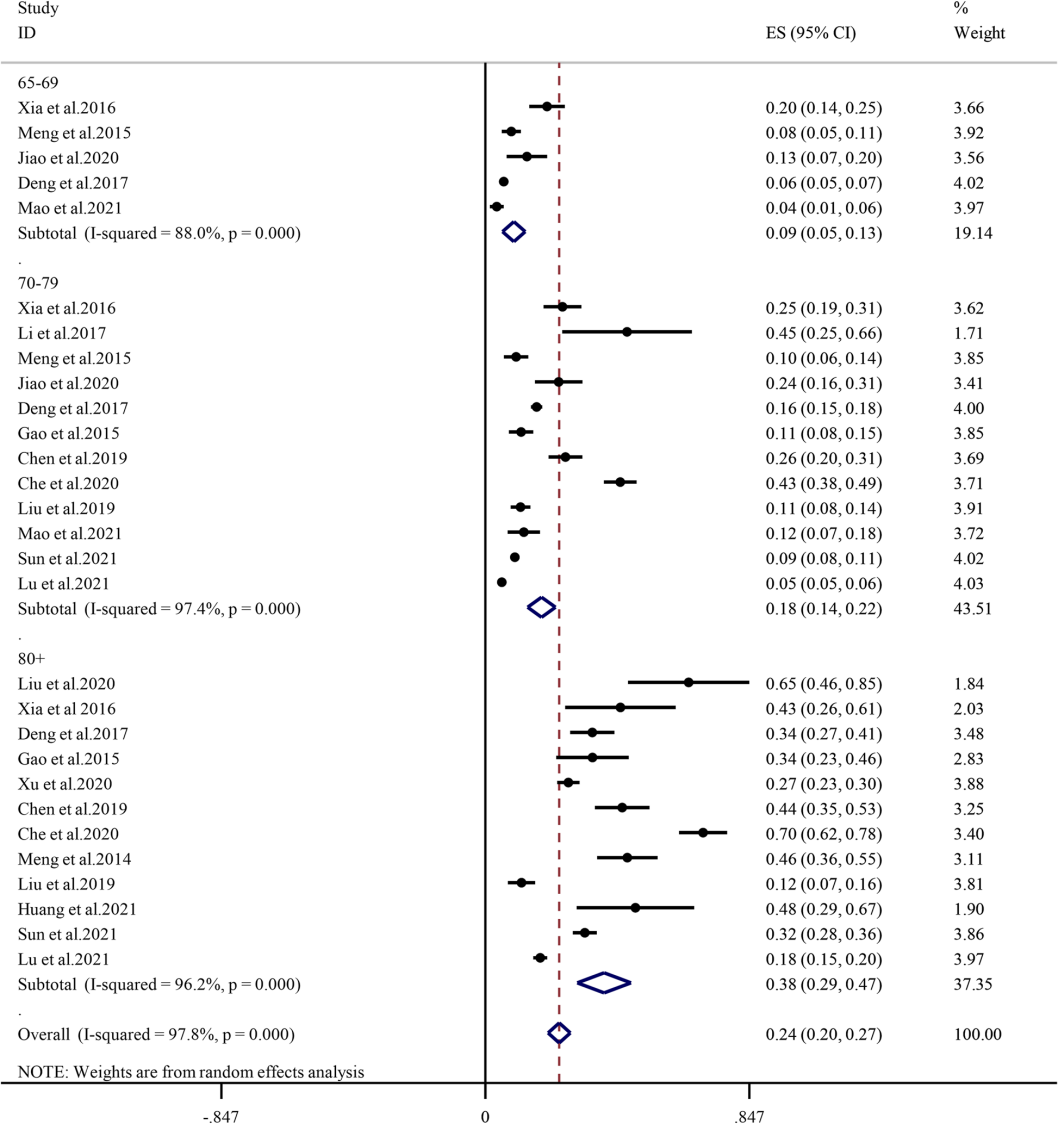
Subgroup analysis of the prevalence of sarcopenia by age

**Fig. 4:**
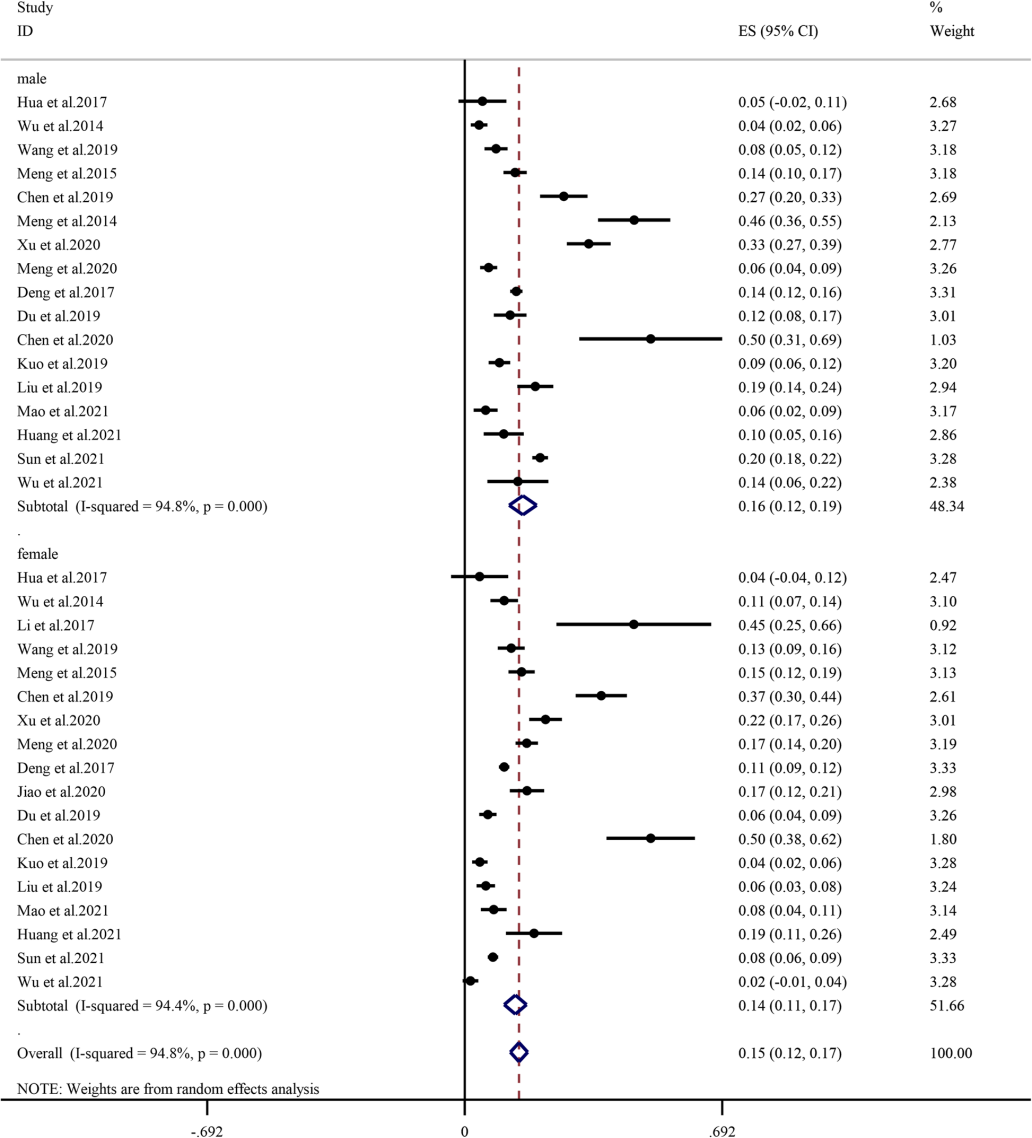
Subgroup analysis of the prevalence of sarcopenia by sex

Subgroup analysis determined by age showed that the prevalence of sarcopenia between 65–69 years, 70–79 years, 80 years and older was 9.1% (95% CI: 5.5%-12.8%, I^2^ = 88.0%, *P* < 0.01), 18.0% (95% CI: 13.6%-22.5%, I^2^ = 97.4%, *P* < 0.01), 38.1% (95% CI: 29.5%-46.7%, I^2^ = 96.2%, *P* < 0.01), respectively (Fig. 3). In all age groups, the prevalence of sarcopenia increased with age; specifically, the prevalence of sarcopenia was lowest (9.1%) in the 65- to 69-year age subgroup and highest (38.1%) in the 80-year and older age subgroups. The overall prevalence was higher among males (15.6%, 95% CI: 11.9%-19.3%, I^2^ = 94.8%, *P* < 0.01) than among females (13.6%, 95% CI: 10.6%-16.6%, I^2^ = 94.4%, *P* < 0.01) (Fig. 4). The prevalence among males (7.7%, 17.3%, 36.2%) and females (4.2%, 12.2%, 32.0%) was consistent with this overall trend (Additional file 3 and Additional file 4).

#### Muscle mass assessment method

**Fig. 5:**
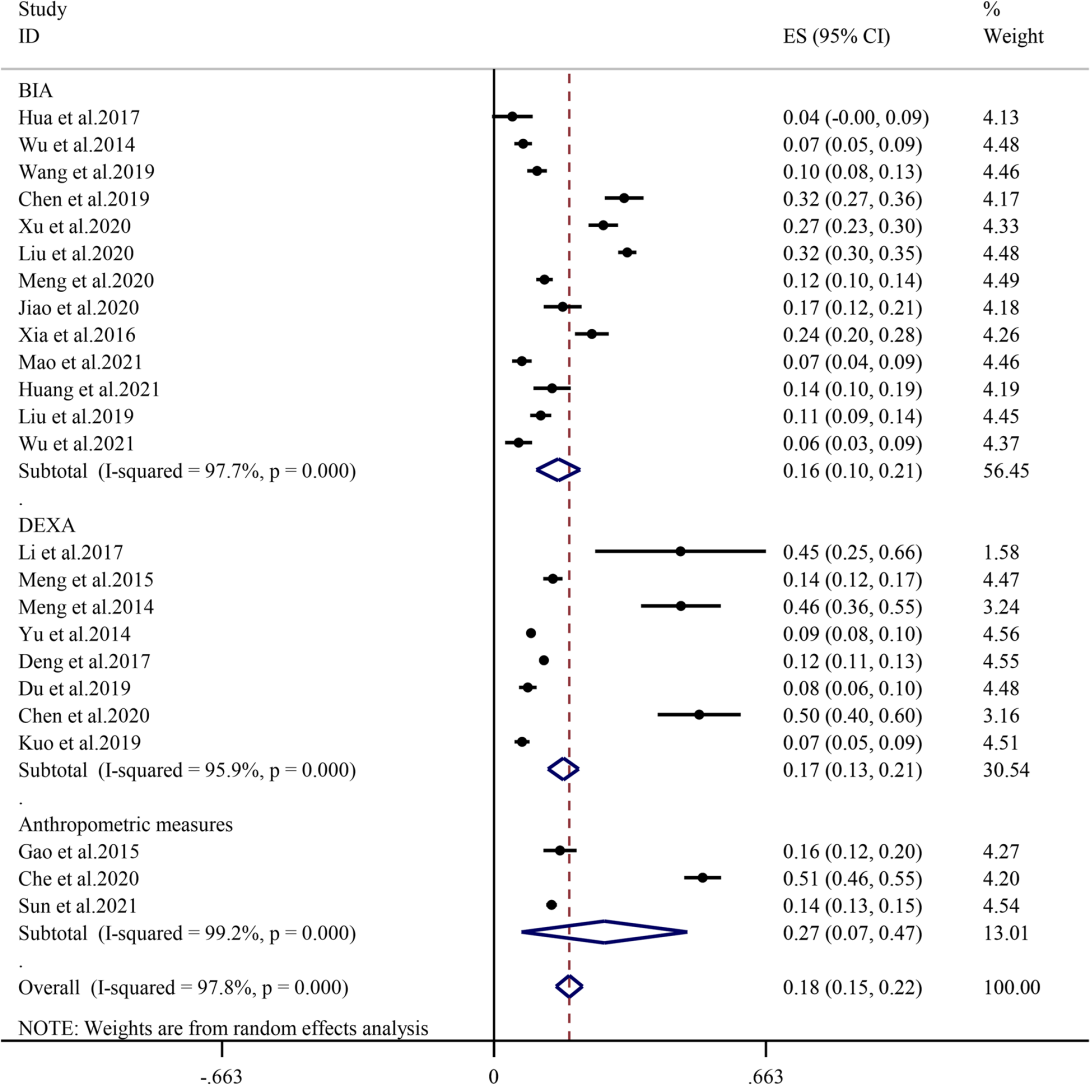
Subgroup analysis of the prevalence of sarcopenia by assessment method

Subgroup analysis by the muscle mass assessment method demonstrated that the prevalence of sarcopenia determined by bioelectric impedance analysis (BIA) and dual-energy X-ray absorptiometry (DEXA) was 16.5% (95% CI: 10.9%-22.0%, I^2^ = 97.7%, *P* < 0.01) and 16.9% (95% CI: 13.2%-20.6%, I^2^ = 95.9%, *P* < 0.01), respectively. The prevalence estimated by anthropometric measures was 26.9% (95% CI: 6.7%-47.1%, I^2^ = 99.2%, *P* < 0.01) (Fig. 5), which was apparently higher than that obtained from studies using BIA and DEXA.

#### Diagnostic criteria

**Fig. 6:**
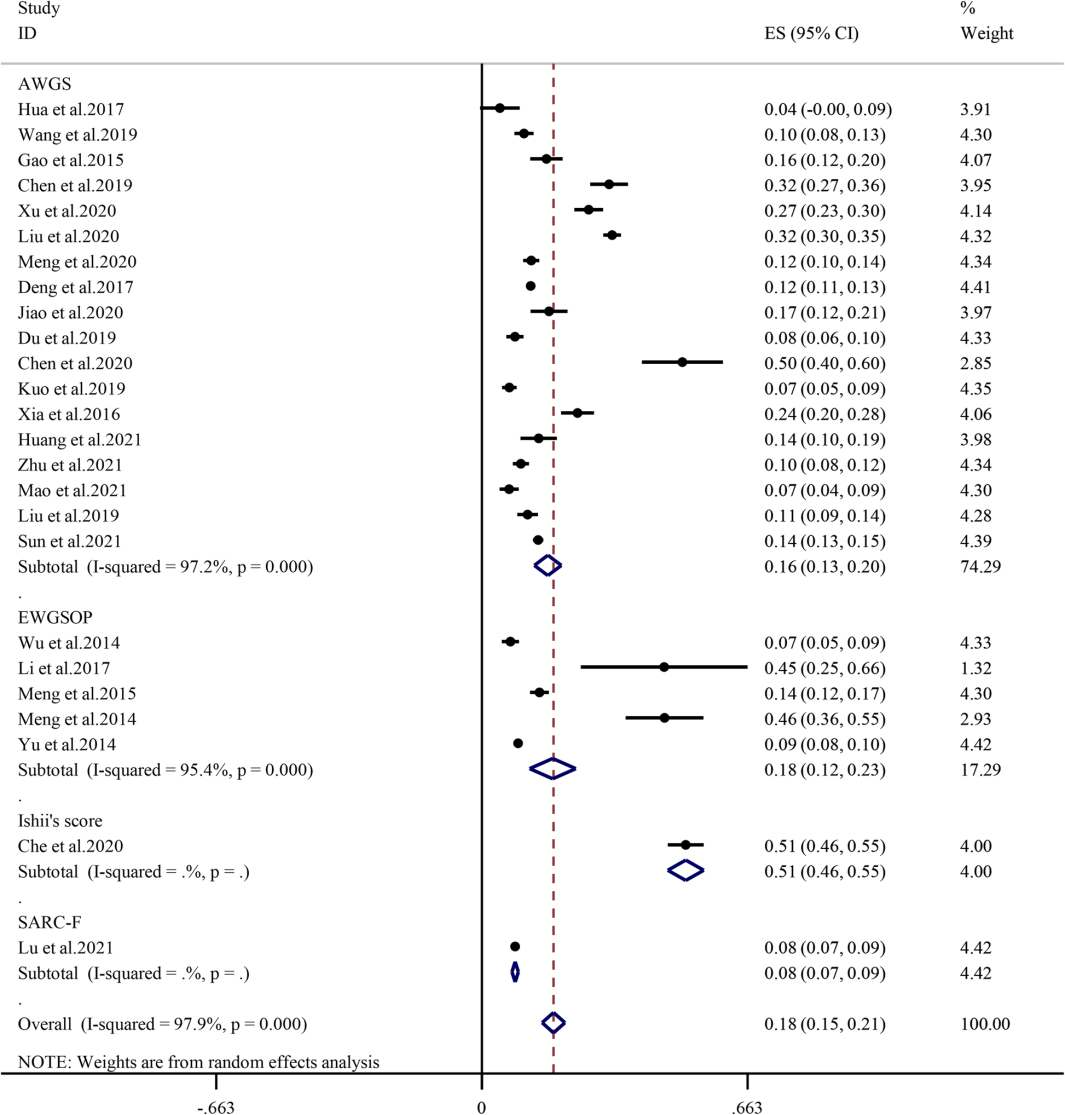
Subgroup analysis of the prevalence of sarcopenia by diagnostic criteria

Subgroup analysis performed by diagnostic criteria revealed that the prevalence of sarcopenia in the AWGS, EWGSOP, Ishii’s score and SARC-F was 16.4% (95% CI: 13.1%-19.8%, I^2^ = 97.2%, *P* < 0.01), 17.7% (95% CI: 11.9%-23.4%, I^2^ = 95.4%, *P* < 0.01), 50.8% (95% CI: 46.4%-55.3%, I^2^ = 0, *P* = 0), and 8.3% (95% CI: 7.5%-9.1%, I^2^ = 0, *P* = 0), respectively (Fig. 6).

#### Area

**Fig. 7:**
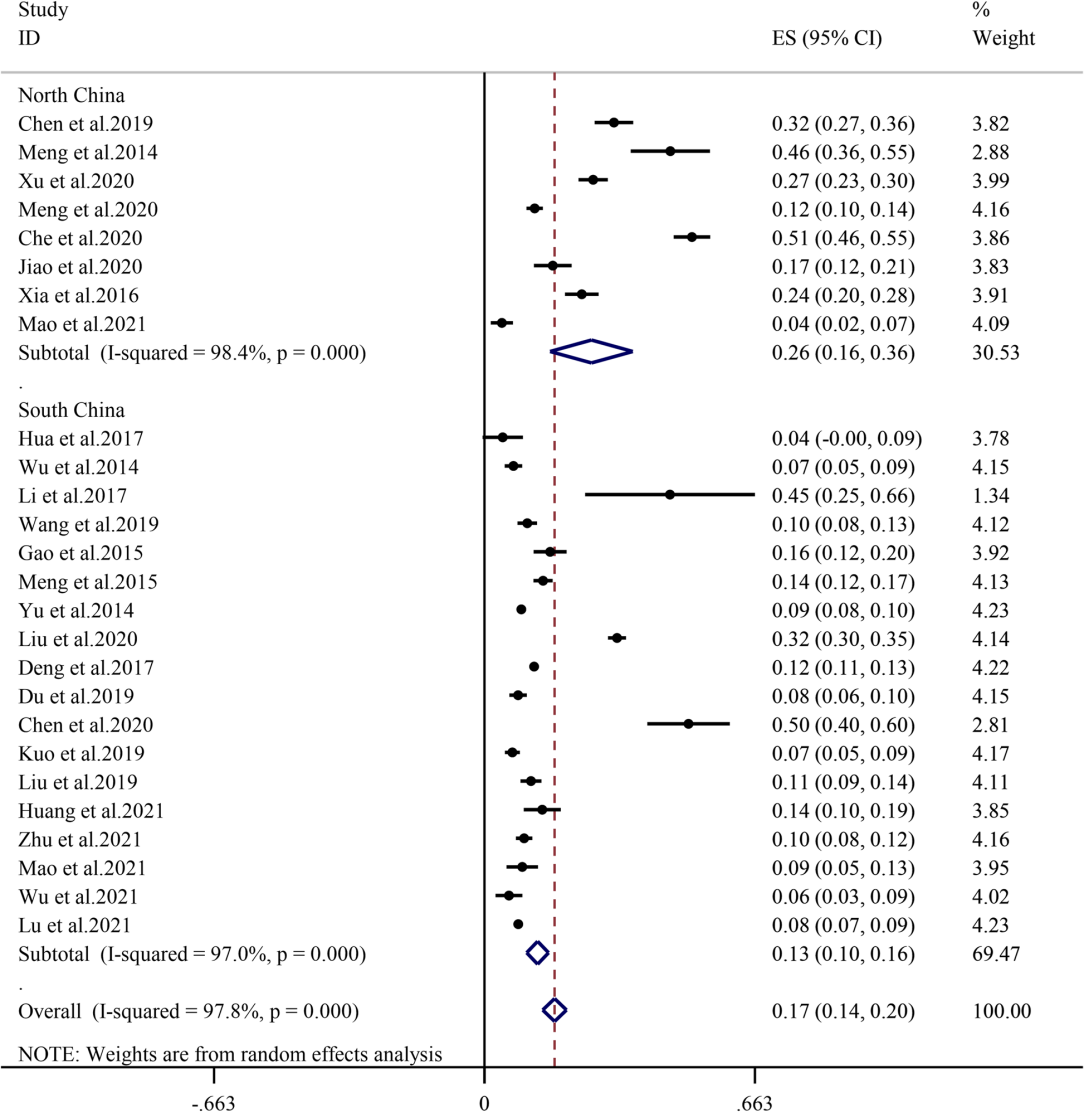
Subgroup analysis of the prevalence of sarcopenia by area

The prevalence of sarcopenia was relatively higher in North China (26.2%, 95% CI: 16.1%-36.3%, I^2^ = 98.4%, *P* < 0.01) than in South China (13.0%, 95% CI: 10.3%-15.7%, I^2^ = 97.0%, *P* < 0.01) (Fig. 7).

### Sensitivity analysis

**Fig. 8:**
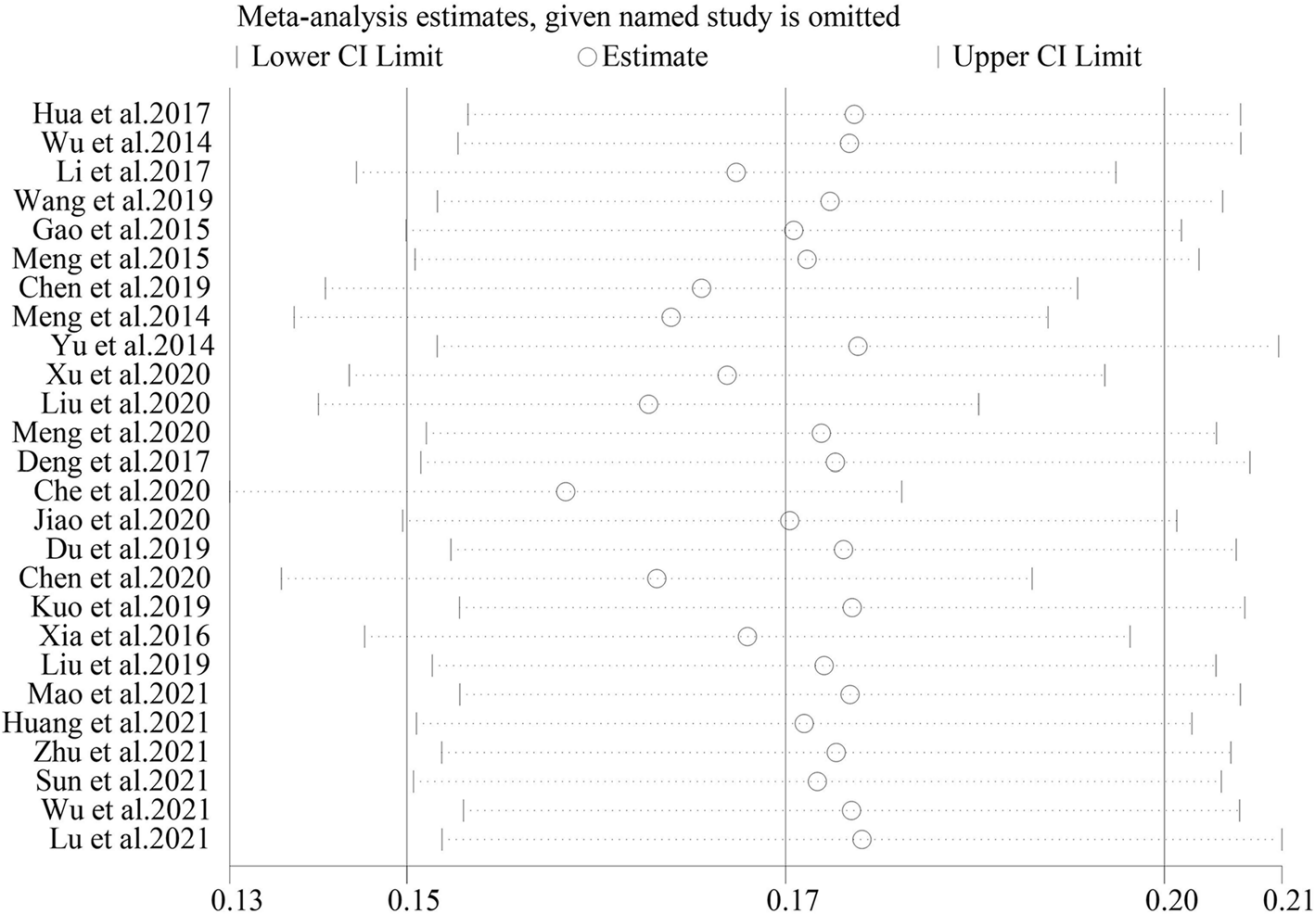
Sensitivity analysis of all included studies

A sensitivity analysis was conducted by removing one study each time and pulling others to determine which study may influence the main effect of the prevalence of sarcopenia in Chinese community-dwelling older adults. No statistically significant changes were found, as shown in Fig. 8.

### Publication bias

**Fig. 9:**
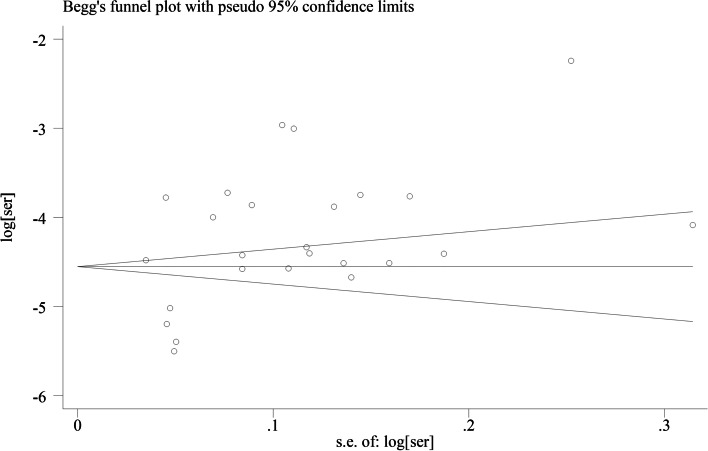
Funnel plot of publication bias of included studies

**Fig. 10:**
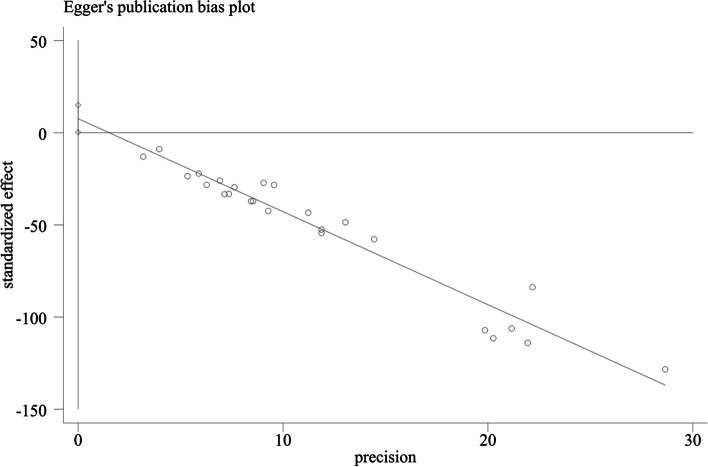
Publication bias plot of included studies by Egger’s test

**Fig. 11:**
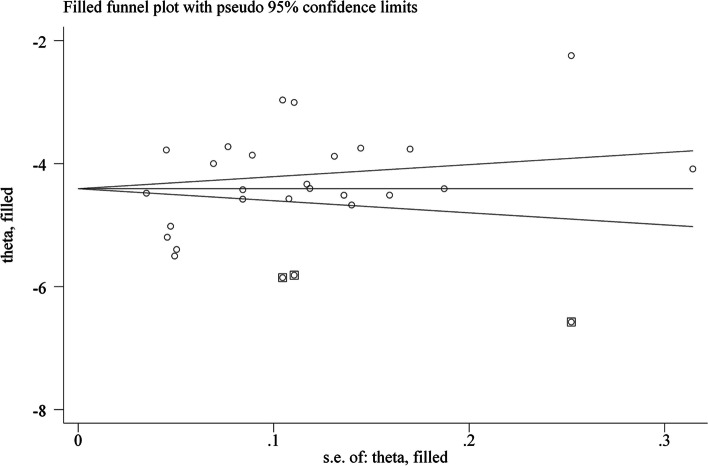
Funnel plot after using the trim and fill method

According to Begg’s test (*P* = 0.624, Fig. 9) and Egger’s test (*P* = 0.043, Fig. 10), statistically significant publication bias was detected in Egger’s test. After using the trim and fill method, the imputed studies produced a symmetrical funnel plot (*P* = 0.548, Fig. 11) with an extra 3 studies filled, and there was no statistically significant publication bias.

## Discussion

This systematic review was conducted to estimate the overall prevalence of sarcopenia (17.4%) in community-dwelling older adults aged over 65 years. We recruited the most studies to characterize the epidemiology of sarcopenia in China. In addition, we compared sarcopenia prevalence between factors that contributed to the great heterogeneity by subgroup analyses of study year, age and sex, muscle mass assessment method, diagnostic criteria, region and area. First, our meta-analyses filled the gap in subgroup analysis by age, and we estimated the age difference in the prevalence of sarcopenia by sex. Second, the prevalence might be overestimated when the muscle mass assessment method was based on anthropometric measures. Third, the AWGS would be better than other standards when measuring the prevalence of sarcopenia among older adults in Chinese communities. Fourth, the prevalence was higher in North China than in South China. This study could provide an all-around comprehension of the prevalence of sarcopenia among Chinese community-dwelling older adults aged over 65 years.

The present study found that the overall prevalence of sarcopenia in Chinese community-dwelling older adults was 17.4%, higher than previous meta-analyses performed by Wu et al. [16] (12%) and Xin et al. [17] (14%). This finding can be explained by the fact that these two studies estimated the overall prevalence of sarcopenia in Chinese community-dwelling older adults aged over 60, but the mean age of our population (65 years and older) was older than these studies. In addition, the prevalence rate is higher than that in other countries. Makizako et al. [48] found that the pooled prevalence of sarcopenia in Japanese community-dwelling older adults aged over 60 was 9.9%. Diz et al. [49] reported an overall prevalence of 17.0% in Brazilians aged over 60; however, this higher prevalence may be due to the inclusion of participants from clinical/hospital and long-term care settings. Shafiee et al.’s [50] meta-analysis showed that the prevalence of sarcopenia among older adults aged 60 years and older in the world is 10% in men and women, so our result is much higher than those of the above studies, which poses a serious challenge to the country.

The meta-analysis results showed that aging was a nonadjustable factor for sarcopenia in both males and females. Subgroup analyses by age showed that the prevalence of sarcopenia between 65–69 years, 70–79 years, 80 years and older was 9.1%, 18.0%, and 37.5%, respectively. Previously, the New Mexico Elder Health Survey study suggested that the prevalence of sarcopenia increased with age; 20% of men between 70–75 years old were affected by sarcopenia, and this increased to 50% of older men aged 80 years or older and 25% and 40% of women between 70–75 years old and 80 years and older, respectively [51]. A 12-year ongoing prospective population-based study conducted in Sweden showed that even participants with no sarcopenia had 10-year probabilities of developing probable sarcopenia and 5.1% [52]. This was consistent with our results that the older the people were, the higher the risk of sarcopenia they would suffer. It must be clarified that not all participants in the included studies could be divided into 65–69, 70–79, 80 years and older subgroups, and the final subgroup analyses based on age were performed only in studies whose participants could be grouped. In addition, men were more likely to suffer sarcopenia than women, which was consistent with previous studies (14% in men vs. 9.11% in women [49]; 13.1–14.9% in men and 11.4% in women [53]). This finding can be explained by the fact that the difference in prevalence between older men and women may be due to lifestyle and smoking or alcohol status [53]. The deleterious influence of lifestyle may be expanded day by day with aging, and all these factors result in sarcopenia. Moreover, Shimokata et al.’s [54] 12-year cohort study also showed that men were more likely to have a significant loss of muscle mass than women.

In the present study, we found that the highest prevalence of sarcopenia defined by the assessment method was the anthropometric measures. As an easy and convenient way to assess the skeletal muscle mass of limbs, it can be used for effective screening for sarcopenia. Although only 3 studies [21, 32, 37] used this measurement tool to define muscle mass, this subjective approach may overestimate the prevalence of sarcopenia. Otherwise, although DEXA was the gold standard to evaluate muscle mass, BIA was more available, cheap, and operable than DEXA and was suitable for extensive screening and diagnosis of sarcopenia in communities and hospitals.

In this meta-analysis, the results of the diagnostic criteria of Ishii’s score estimated the highest prevalence [32] (50.8%); however, there were only two studies included in the subgroup based on Ishii’s score and SARC-F. The pooled results might be not reliable and need to be interpreted with caution. However, Li et al.’s [55] study verified that Ishii’s score had a high screening value among community-dwelling older adults. This result reminded us that the prevalence of sarcopenia among Chinese community-dwelling older adults may vary in terms of different diagnostic criteria. In addition, the cutoff values used in different diagnostic criteria also influenced the prevalence of sarcopenia. In Xia et al.’s [43] study, this relative skeletal muscle mass index (RSMI) value was slightly higher among women, which may underestimate the prevalence of sarcopenia among older women. Nevertheless, 5 studies [25, 28, 30, 34, 40] used the EWGSOP as the diagnostic criteria, and those studies cutoff values of muscle mass and muscle strength were different from each other, which may influence the overall prevalence. For comparability among Asian community-dwelling older adults’ studies, it would be better to choose the AWGS, which has the same cut point value to diagnose sarcopenia, to diagnose the prevalence of sarcopenia in community-dwelling older adults.

The subgroup analysis of region or area showed that the prevalence of sarcopenia in North China (26.9%) was higher than that in South China (13.0%). To date, this is the first study to analyze the area difference in the prevalence of sarcopenia in China. Although Mao et al.’s [39] study separated their area into a northern city and a southern city, the result was inconsistent with our study. The high prevalence in North China in our meta-analysis may be due to one of the studies evaluating the prevalence in 80-year-old and old men living in Beijing [30]. In addition, we inferred that this prevalence may be affected by the climate and diet patterns caused by geographical differences. Northern older adults may have less time to participate in outdoor physical activity due to the climate influence, and in their daily diet, cereals, eggs, beans and soy products were consumed more, while southern older adults consumed more aquatic products such as rice or fish [56]. They were naturally at greater risk of malnutrition and sarcopenia due to dietary preferences. Considering China as a multiethnic and large country, our findings should be further verified by future meta-analyses focusing on sarcopenia-associated risk factors, including diet structure, physical activity, climate, or diseases such as osteoarthritis, based on geography.

There are also limitations in this study. First, limited to the characteristics of individual studies and the situation of sarcopenia prevalence varying greatly from city to city, the heterogeneity among the included studies was strong. Second, among the 26 included studies, it only covered some province rather than the whole country, so the results cannot be generalized to reveal the overall prevalence of community-dwelling older adults in China. Third, due to the lack of a clear division of geographical location in the included studies, it was not possible to make a detailed subgroup analysis among urban and rural areas. Finally, some included studies did not provide needed information, such as the prevalence among different sexes and ages, so we could not analyze the source of heterogeneity.

## Implications

The results of this meta-analysis revealed a serious situation of the overall prevalence of sarcopenia in Chinese community-dwelling older adults aged over 65 (17.4%), which should attract more attention from sports and public health departments. We also advise that future studies use BIA and AWGS to evaluate the prevalence of sarcopenia, which is more suitable for Chinese community-dwelling older adults. Considering that the prevalence of sarcopenia differs by region and area, the public health sectors need to take specific measures or publish targeted health-care policies to prevent the exacerbation of this situation.

## Supplementary Information

**Additional file 1: Table S1. **Characteristics of the included studies.

**Additional file 2: Figure S1. **Subgroup analysis of the prevalence of Sarcopenia by study year.

**Additional file 3: Figure S2. **The prevalence of sarcopenia in males by age group.

**Additional file 4: Figure S3.** The prevalence of sarcopenia in females by age group.

## Data Availability

All data generated or analyzed during this study are included in this published article and its supplementary information files.

## Abbreviations

ASM: Appendicular Skeletal Muscle
AWGS: Asian Working Group for Sarcopenia
BIA: Bioelectric Impedance Analysis
CNKI: China National Knowledge Infrastructure
DEXA: Dual Energy X-ray Absorptiometry
EWGSOP: European Working Group on Sarcopenia
FNIH: Foundation for the National Institutes of Health
GRADE: Grading of Recommendations Assessment, Development and Evaluation
ICD-10-CM: International Classification of Diseases Clinical Modification
IWGS: International Working Group on Sarcopenia
NA: Not Available
PRISMA: Preferred Reporting Items for Systematic Reviews and Meta Analyses
RSMI: Relative Skeletal Muscle mass Index
SMI: Skeletal Muscle Index
SPPB: Short Physical Performance Battery
TUG: Time up and Go test
WHO: World Health Organization
