# Supplementary material for: Prevalence of sarcopenia in Chinese community-dwelling elderly: a systematic review
Source: BMC Public Health. 2022 Sep 8;22:1702. doi: 10.1186/s12889-022-13909-z (PMC9454186; doi:10.1186/s12889-022-13909-z)
Supplement: Supplementary file 1 — Additional file 1: Table S1. Characteristics of the included studies. [file 12889_2022_13909_MOESM1_ESM.docx]

Additional file 1: Table S1 Characteristics of the included studies

| Study  (Author & year) | Area | Study design | Age  (years) | Defining  criteria | Assessment method and cutoff values | | | Sample size | | | Sarcopenia Prevalence (%) | | |
| --- | --- | --- | --- | --- | --- | --- | --- | --- | --- | --- | --- | --- | --- |
|  |  |  |  |  | Muscle mass | Muscle strength  (handgrip strength) | Muscle performance (gait speed) |  |  |  |  |  |  |
|  |  |  |  |  |  |  |  | All | Male | Female | all | Male | Female |
| Hua et al  2017 | Shanghai | Cross-sectional | ≥65 | AWGS | BIA  Male＜7.0 kg/m^2^  Female＜5.7 kg/m^2^ | Male＜26 kg  Female＜16 kg | 0.8 m/s | 67 | 42 | 25 | 4.48% | 4.76% | 4.00% |
| Wu et al  2014 | Kaohsiung | Cross-sectional | ≥65 | EWGSOP | BIA  Male＜7.0 kg/m^2^  Female＜5.7 kg/m^2^ | Male ≤29 kg(BMI≤24), ≤30 kg(24.1＜BMI≤28), ≤32 kg(BMI＞28)  Female≤17 kg(BMI≤23), ≤17.3 kg (23.1＜BMI≤26), ≤18 kg(26.1＜BMI＜29), ≤21 kg(BMI＞29) | 0.8 m/s | 549 | 285 | 264 | 7.10% | 3.86% | 10.61% |
| Li et al  2017 | Shanghai | Cross-sectional | 70-79 | EWGSOP | DEXA  female<5.5 kg/m^2^ | Female<20 kg | 0.8 m/s | 22 | NA | 22 | 45.45% | NA | 45.45% |
| Wang  2019 | Chengdu | Cross-sectional | ≥60 | AWGS | BIA  Male<7.0 kg/m^2^, female<5.7 kg/m^2^ | Male<26 kg  Female<18 kg | 0.8 m/s | 629 | 309 | 320 | 10.49% | 8.41% | 12.50% |
| Gao et al  2015 | Chengdu, Suining | Cross-sectional | ≥60 | AWGS | Anthropometric Measures  CC<31 cm | Male<26 kg  Female<18 kg | 0.8 m/s | 317 | NA | NA | 16.09% | NA | NA |
| Meng et al  2015 | Taichung | Cross-sectional | ≥65 | EWGSOP | wSMI: 27.1% for male and 23.7% for female.  Or hSMI: Male<6.39 kg/m^2^, female<4.84 kg/m^2^ | Male<30 kg  Female<20 kg | 0.8 m/s | 857 | 458 | 399 | 14.35% | 13.54% | 15.29% |
| Chen et al  2019 | Beijing | Cross-sectional | 60-89 | AWGS | BIA  Male<7.0 kg/m^2^, female<5.7 kg/m^2^ | Male<28 kg  Female<18 kg | 0.8 m/s | 372 | 184 | 188 | 31.72% | 26.63% | 36.70% |
| Meng et al  2014 | Beijing | Cross-sectional | ≥80 | EWGSOP | ASM index or SMI  Male<6.85 kg/m^2^, | Male<22.4 kg | 0.8 m/s | 101 | 101 | NA | 45.54% | 45.54% | NA |
| Yu et al  2014 | Hong Kong | Cohort | ≥65 | EWGSOP | ASM index  Male<6.52 kg/m^2^,  female<5.44 kg/m^2^ | Male<28 kg  Female<18 kg | 0.8 m/s | 4000 | 2000 | 2000 | 9.03% | NA | NA |
| Xu et a;  2020 | Beijing | Cross-sectional | ≥80 | AWGS | BIA  Male<7.0 kg/m^2^, female<5.7 kg/m^2^ | Male<26 kg  Female<18 kg | 0.8 m/s | 582 | 246 | 336 | 26.63% | 33.33% | 21.73% |
| Liu et al  2020 | Yunnan, Guizhou, Sichuan, Xinjiang | Cross-sectional | ≥50 | AWGS | BIA  Male<7.0 kg/m^2^, female<5.7 kg/m^2^ | Male<26 kg  Female<18 kg | 0.8 m/s | 1712 | NA | NA | 32.48% | NA | NA |
| Meng et al  2020 | Xinxiang | Cross-sectional | ≥65 | AWGS | BIA  Male<7.0 kg/m^2^, female<5.7 kg/m^2^ | Male<28 kg,  Female<18 kg | 0.8 m/s | 1024 | 436 | 588 | 12.30% | 6.42% | 16.67% |
| Deng et al  2017 | Shenzhen | Cross-sectional | ≥65 | AWGS | DEXA  Male<7.0 kg/m^2^, female<5.4 kg/m^2^ | Male<26 kg  Female<18 kg | 0.8 m/s | 3494 | 1673 | 1821 | 12.16% | 13.87% | 10.60% |
| Che et al  2020 | Urumqi | Cross-sectional | ≥60 | Ishii’s score | Anthropometric Measures | NA | NA | 478 | NA | NA | 50.84% | NA | NA |
| Jiao et al  2020 | Tianjin | Cross-sectional | 60-89 | AWGS | BIA  Female<5.7 kg/m^2^ | Female<18 kg | 0.8 m/s | 251 | NA | 251 | 16.73% | NA | 16.73% |
| Du et al  2020 | Shanghai | Cross-sectional | 65-89 | AWGS | DEXA  Male<6.66 kg/m^2^, female<5.24 kg/m^2^ | Male<24.8 kg  Female<15 kg | 0.8 m/s | 631 | 213 | 418 | 12.20% | 12.21% | 6.22% |
| Chen et al  2020 | Taipei | Cross-sectional | ≥65 | AWGS | DEXA  Male<7.0 kg/m^2^, female<5.7 kg/m^2^ | Male<26 kg  Female<18 kg | 0.8 m/s | 94 | 26 | 68 | 50.00% | 50.00% | 50.00% |
| Kuo et al  2019 | Yilan | Cross-sectional | ≥65 | AWGS | DEXA  Male<7.0 kg/m^2^, female<5.4kg/m^2^ | Male<26 kg  Female<18 kg | 0.8 m/s | 731 | 386 | 345 | 6.84% | 9.33% | 4.06% |
| Xia et al  2016 | Beijing | Cross-sectional | ≥60 | AWGS | BIA  Male<7.0 kg/m^2^, female<5.8 kg/m^2^ | Male<26 kg  Female<18 kg | 0.8 m/s | 411 | NA | NA | 23.84% | NA | NA |
| Liu et al  2019 | Shanghai | Cross-sectional | ≥60 | AWGS | BIA  Male<7.0 kg/m^2^, female<5.7 kg/m^2^ | Male<26 kg  Female<18 kg | 0.8 m/s | 588 | 253 | 335 | 11.39% | 18.97% | 5.67% |
| Mao et al  2021 | Beijing, Chengdu, Guangzhou, Wuhu, Xuchang, Lanzhou, Jilin, Chenzhou | Cross-sectional | ≥65 | AWGS | BIA  Male<7.0 kg/m^2^, female<5.7 kg/m^2^ | Male<26 kg  Female<18 kg | 0.8 m/s | 427 | NA | NA | 6.79% | 5.61% | 7.79% |
| Huang et al  2021 | Zhejiang | Cross-sectional | ≥60 | AWGS | BIA  Male<7.0 kg/m^2^, female<5.7 kg/m^2^ | Male<26 kg  Female<18 kg | 0.8 m/s | 226 | 124 | 102 | 14.16% | 10.48% | 18.63% |
| Zhu et al  2021 | Ningbo | Cross-sectional | ≥65 | AWGS | SMI  Male<7.0 kg/m^2^, female<5.7 kg/m^2^ | Male<26 kg  Female<18 kg | 0.8 m/s | 823 | 339 | 484 | 9.72% | NA | NA |
| Sun et al  2021 | China | Cross-sectional | ≥60 | AWGS | Lee’s formula  Male<7.0 kg/m^2^, female<5.4 kg/m^2^ | Male<26 kg  Female<18 kg | 0.8 m/s | 2759 | 1401 | 1358 | 14.03% | 20.27% | 7.58% |
| Wu et al  2021 | Southern Taiwan | Cross-sectional | ≥65 | NA | NA | Male<26 kg  Female<18 kg | 0.8 m/s | 200 | 70 | 130 | 6.00% | 14.29% | 1.54% |
| Lu et al  2021 | Shanghai | Cross-sectional | ≥50 | SARC-F | NA | NA | NA | 4576 | NA | NA | 8.28% | NA | NA |

Note: AWGS, Asia Working Group for Sarcopenia; ASM, appendicular skeletal muscle; BIA, bioelectrical impedance analysis; CC, calf circumference; DEXA, dual-energy X-ray absorptiometry; EWGSOP, European Working Group on Sarcopenia in Older People; NA, not available; SMI, skeletal muscle index
