# Supplementary figures and images for: Prevalence of sarcopenia in Chinese community-dwelling elderly: a systematic review
Source: BMC Public Health. 2022 Sep 8;22:1702. doi: 10.1186/s12889-022-13909-z (PMC9454186; doi:10.1186/s12889-022-13909-z)

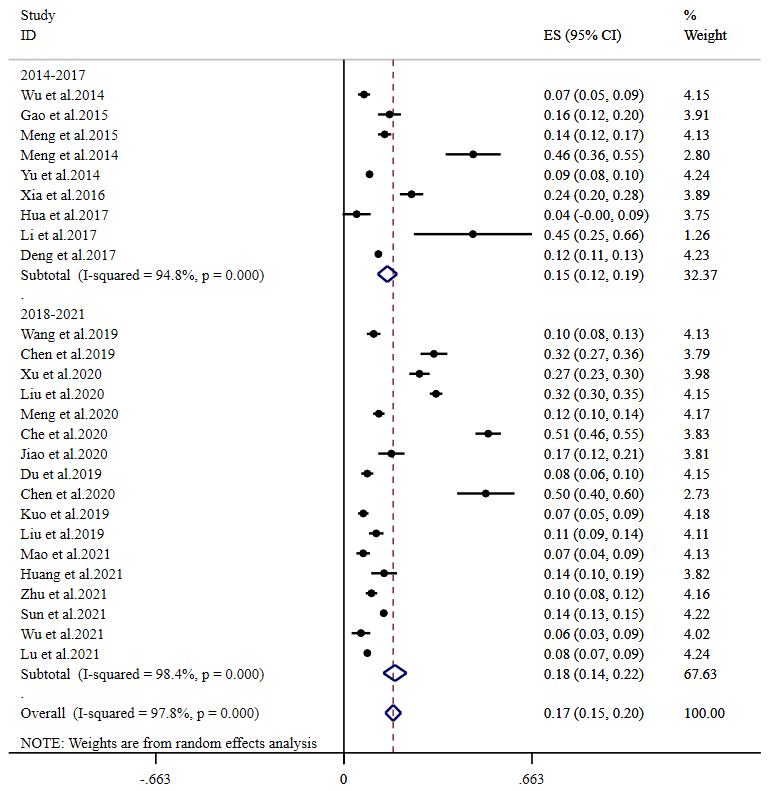


Additional file 2: Figure S1.

Supplement: Supplementary file 2 — Additional file 2: Figure S1. Subgroup analysis of the prevalence of Sarcopenia by study year. [file 12889_2022_13909_MOESM2_ESM.docx]

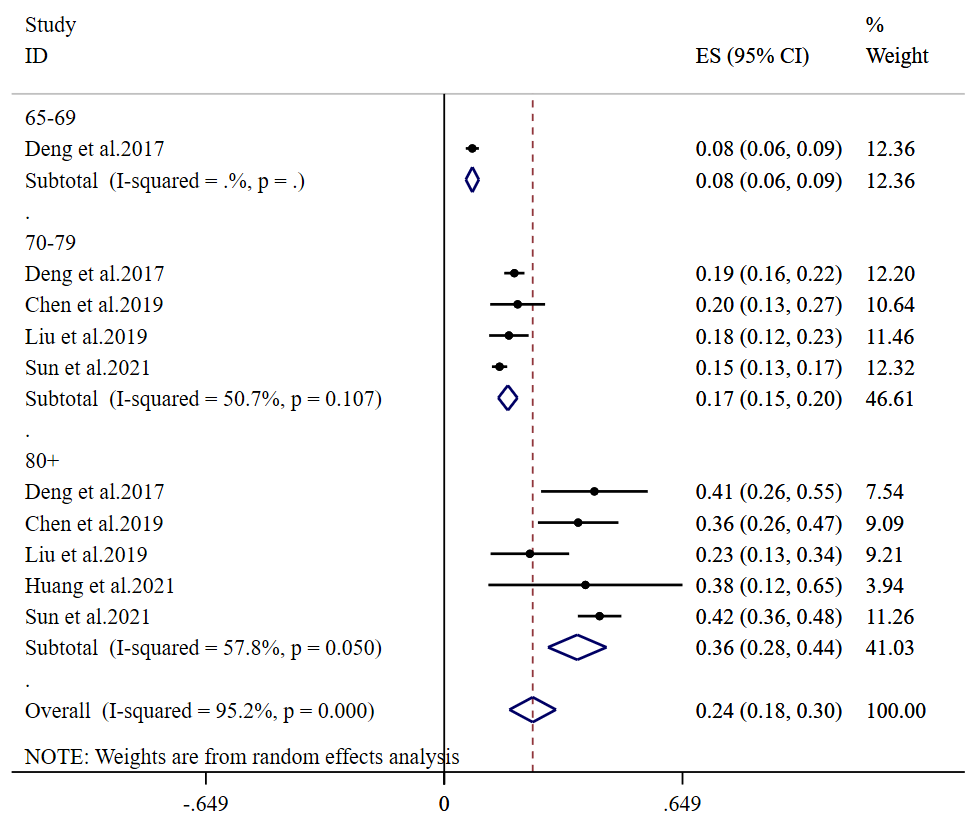


Additional file 3: Figure S2.

Supplement: Supplementary file 3 — Additional file 3: Figure S2. The prevalence of sarcopenia in males by age group. [file 12889_2022_13909_MOESM3_ESM.docx]

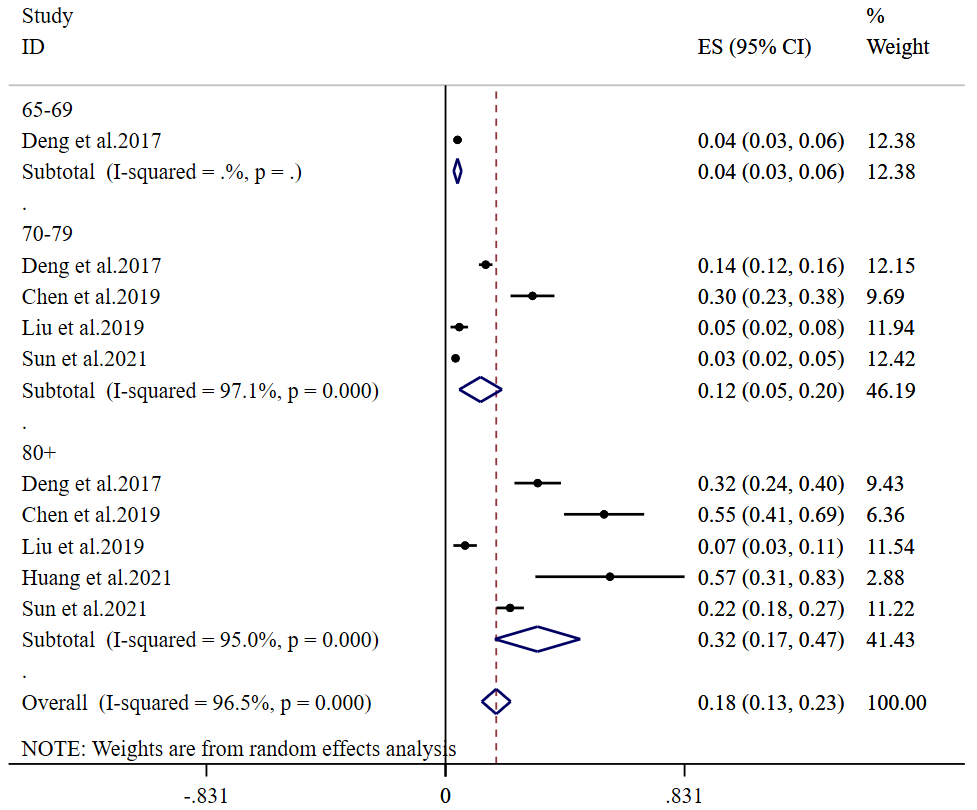


Additional file 4: Figure S3.

Supplement: Supplementary file 4 — Additional file 4: Figure S3. The prevalence of sarcopenia in females by age group. [file 12889_2022_13909_MOESM4_ESM.docx]
